# Rice Wine Fermentation: Unveiling Key Factors Shaping Quality, Flavor, and Technological Evolution

**DOI:** 10.3390/foods14142544

**Published:** 2025-07-21

**Authors:** Baoyu Peng, Haiyang Huang, Jingjing Xu, Yuan Xin, Lang Hu, Lelei Wen, Li Li, Jinwen Chen, Yu Han, Changchun Li

**Affiliations:** 1 Hubei Key Laboratory of Resource Utilization and Quality Control of Characteristic Crops, College of Life Science and Technology, Hubei Engineering University, Xiaogan 432000, China; pbaoyu@163.com (B.P.); wenlelei@163.com (L.W.); lili2023new@163.com (L.L.); 2 College of Life Science and Technology, Hubei Engineering University, Xiaogan 432000, China; toto741@163.com (H.H.);; 3 College of Food Science & Technology, Huazhong Agricultural University, Wuhan 430070, China

**Keywords:** rice wine, fermentation technology, microbial community, flavor regulation, quality control, traditional brewing, raw materials

## Abstract

Rice wine, as a traditional fermented beverage, has its quality and flavor influenced by a combination of multiple factors. This review provides an overview of the key aspects of rice wine production, including raw material selection and processing, the regulation of quality by brewing techniques, the mechanisms of microbial community interaction during fermentation, and the types and formation mechanisms of major compounds in rice wine (including flavor compounds and non-volatile components). The study highlights that different raw materials and processing methods significantly impact the fundamental flavor profile of rice wine, while fermentation conditions and dynamic changes in microbial communities determine its flavor complexity and stability. Additionally, this review examines various factors affecting the quality and flavor of rice wine, such as fermentation environment, microbial metabolism, and control of harmful substances, and summarizes modern research and technological advancements, emphasizing the potential of digital and intelligent technologies in enhancing the quality and safety of rice wine. Finally, future research directions are proposed to promote modernization and quality improvement of the rice wine industry.

## 1. Introduction

### 1.1. Definition and Cultural Context of Rice Wine

Rice wine, also known as mijiu, jiuniang, láo zào, glutinous rice wine, or sweet fermented rice, is a traditional low-alcohol beverage (typically containing 3–12% alcohol by volume) brewed from water and cereal grains such as rice, glutinous rice, red rice, and black rice through processes including soaking, steaming, inoculation with qu (also refer as fermentation starter or koji), saccharification, and fermentation [1,2]. With a brewing history spanning millennia in China, it has gained widespread popularity across East Asia and parts of Southeast Asia [3,4,5,6,7]. Deeply embedded in cultural practices such as festivals, rituals, and family gatherings, rice wine symbolizes auspiciousness, reunion, and harvest. Over centuries, its brewing techniques have evolved into distinct regional styles, while its cultural significance remains a cherished heritage for preservation and promotion [8].

As one of the world’s oldest alcoholic beverages, rice wine stands alongside beer, wine, and yellow wine as a gem of traditional fermentation culture. Renowned for its unique flavor profile, low alcohol content, and rich nutritional components—including amino acids, polyphenols, vitamins, trace elements, and functional oligosaccharides—it holds enduring appeal among Chinese consumers [9]. In recent years, market demand for rice wine has surged. According to the National Bureau of Statistics, China’s yellow wine production reached approximately 3.5 million kiloliters in 2022, while glutinous rice wine output hit 2 million kiloliters, solidifying the nation’s status as a leading producer and consumer of rice wine [10].

### 1.2. Geographical Distribution and Regional Characteristics

Chinese rice wine, a historically rich fermented beverage, showcases diverse flavor profiles shaped by regional environments and brewing techniques. Predominantly produced south of the Qinling-Huaihe line, it spans four main regions—Guangdong-Guangxi, Hunan-Hubei, Yunnan-Guizhou-Sichuan, and coastal areas—tied to rice cultivation zones [10,11,12]. Regional variations yield distinct styles: Jiangsu-Zhejiang’s sweet Jiuniang [13,14,15,16], Sichuan-Guizhou’s mi láo zào [17,18], Fujian’s Hong-qu rice wine [19], and Hakka niangjiu [6,20]. Zhejiang’s Shaoxing Jianhu rice wine stands out historically, with methods documented in *Qimin Yaoshu* and refined during the Song Dynasty [21].

Hubei Xiaogan rice wine, a Hunan-Hubei staple, uses local glutinous rice and Fengwo qu [22,23], yielding a clear, fragrant, and sweet beverage. Granted EU market access via the China-Europe Geographical Indications Agreement in 2020, it joined the “Belt and Road” promotion list in 2024, highlighting its cultural and technical value. In Yunnan-Guizhou-Sichuan, Dazhu láo zào—crafted from regional glutinous rice—earned National Geographical Indication status in 2011 for its rich texture [17]. Guangdong Shunde’s red rice wine, embodying Lingnan terroir, uses Xijiang-Beijiang water and traditional techniques with local rice and red Jiuqu [15,24].

Across Asia (10° N–45° N latitude [11]), rice wine variants like Japanese sake [7], Korean makgeolli [25], and Vietnamese rice wine [4] share glutinous rice bases but differ in ingredients, methods, and traditions [26]. With millennia of history, Chinese rice wine thrives for its flavor, nutrition, and cultural significance [2]. Modern research has shifted toward scientific, precise production [27,28,29], and this paper reviews fermentation processes, microbial ecology, flavor mechanisms, and health benefits to support sustainable industry growth.

## 2. Brewing Process of Rice Wine

**Figure 1:**
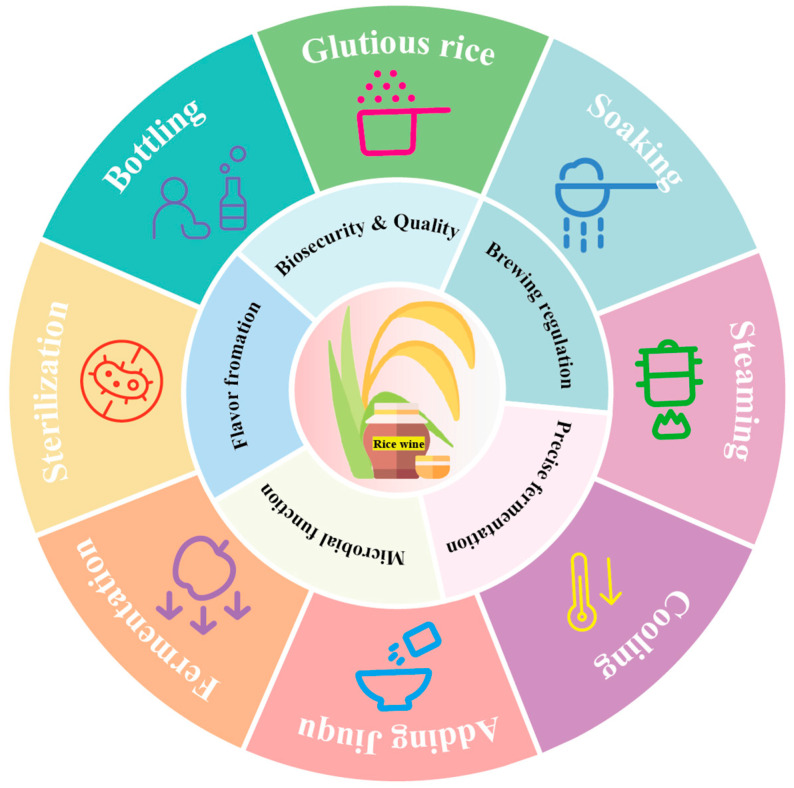
The traditional rice wine brewing process and key elements: brewing regulation, fermentation control, microbial functions, flavor formation, bio-safety, and quality.

The traditional brewing process of rice wine primarily includes rice soaking, steaming, cooling and qu inoculation, fermentation, pressing and clarification, and sterilization [2] (Figure 1).

**Ricesoaking:** High-quality glutinous rice is selected, thoroughly rinsed to remove bran, impurities, and floating debris until the rinsing water becomes clear. The rice is then soaked for 6–10 h (adjusted based on water temperature), ensuring the integrity of the grains.

**Steaming:** The soaked rice is drained and placed in a steamer, where it is cooked over high heat until fully gelatinized but not mushy or burnt.

**Cooling and qu inoculation:** The steamed rice is cooled to 28–30 °C, after which an appropriate amount of qu is evenly mixed into the rice. Qu typically contains a diverse microbiota, including molds such as *Aspergillus oryzae*, yeasts like *Saccharomyces cerevisiae*, and lactic acid bacteria like *Lactobacillus* species, which initiate the fermentation process.

**Fermentation:** The inoculated rice is transferred to fermentation vessels for microbial activity. During this stage, the microorganisms in the qu break down the starches into fermentable sugars, which are then converted into alcohol and other flavor compounds.

**Pressing, clarification, and sterilization:** After fermentation is complete, the processing methods vary depending on the intended use (such as for home consumption or as a commercial beverage). Some producers choose to press and filter the fermented mash to remove the residual lees, followed by clarification and sterilization in sequence [28]. In contrast, other producers may opt to sterilize the mash directly after fermentation, ultimately obtaining the finished rice wine that contains glutinous rice components [30].

In rice wine production, exogenic additives such as fruit pulp, traditional Chinese medicinal herbs, enzymes, and microbial inoculants are used to enhance flavor, nutritional value, and fermentation efficiency. These carefully selected additives improve sensory characteristics and meet consumer preferences for health benefits.

### 2.1. Traditional Preparation of Qu

Qu, also known as koji, is critical for fermentation, with two main types. Small Qu, made from rice/rice flour, is formed into small balls/cakes (sometimes with herbs/spices). Prepared by mashing rice into paste, shaping, drying on bamboo sieves, it is often inoculated with prior qu powder for specific microbes. Big Qu, wheat-based and brick-shaped, is made by pressing grain paste into molds, partial sun-drying, and incubating in darkness; moisture and brick positions are adjusted for uniform microbial growth.

### 2.2. Traditional Solid-State Fermentation

Currently, rice wine production primarily relies on traditional solid-state fermentation (SSF), a method where microorganisms ferment in a low-moisture solid substrate [31]. SSF is integral to producing rice wine and involves several key steps.:

**Preparation of raw materials:** High-quality rice is steamed to activate starch conversion into fermentable sugars.

**Inoculation with qu:** Steamed rice is mixed with qu, a fermentation starter rich in molds (e.g., *Aspergillus* spp.), yeasts (e.g., *Saccharomyces* spp.), and bacteria (e.g., *Lactobacillus* spp.).

**Fermentation conditions:** The mixture is kept at 28–30 °C, allowing microorganisms to convert sugars into alcohol over several days to weeks.

This method is favored for its low raw material costs, wide availability of sources, simple technology, and the fact that open mixed-culture fermentation does not require strict aseptic conditions. However, it also has limitations such as low production efficiency and difficulty in precisely controlling product quality [27]. The fermentation process typically involves two main stages: lactic acid fermentation and alcoholic fermentation. Lactic acid fermentation, driven by lactic acid bacteria (LAB), usually lasts 24–48 h. During this stage, LAB such as *Lactobacillus plantarum*, *Lactobacillus paracasei*, and *Leuconostoc mesenteroides* convert sugars into lactic acid, lowering the pH and creating a favorable environment for subsequent fermentation steps. This is followed by alcoholic fermentation, which generally takes 3–7 days, during which yeasts like *Saccharomyces cerevisiae* convert fermentable sugars into ethanol and carbon dioxide, producing alcohol and contributing to the flavor profile.

Given these limitations, some scholars both domestically and internationally have begun to explore the use of liquid fermentation for rice wine production [32,33]. Investigations have demonstrated that the optimization of rice wine brewing protocols can be achieved through meticulous studies on raw material pretreatment, including aspects such as processing precision and the extent of grinding [34,35]. Researchers have also developed new rice wine brewing processes by using enzymatic preparations to replace traditional qu cultures for liquefaction and saccharification, combined with pure yeast fermentation [36,37,38,39,40].

### 2.3. Criteria for High-Quality Rice Wine

Rice wine production and quality are regulated by various national and international standards to ensure safety and consistency. For example, Cambodia’s CTR 150:2024 sets a maximum methanol level of 2000 mg per liter of alcohol. China’s GB/T 13662–2018 and GB/T 27588-2011 provide methods for determining total acid and ester content, while GB5009.124–2016 measures free amino acids. Current rice-wine regulation is anchored in the dual framework of NY/T 1885-2017’s green, graded risk governance and QB/T 8152-2025’s β-phenethyl-olfactory/sterilization bifurcation, ensuring convergent oversight of safety and sensory attributes.South Korea’s standards for Takju and Yakju specify limits on ethanol, total acid, and methanol content. These standards collectively ensure that rice wine meets specific quality and safety criteria.

## 3. Raw Material Selection and Processing

### 3.1. Rice Variety Selection

Chinese rice wine brewing hinges on meticulous rice variety selection, with glutinous rice traditionally favored for its high amylopectin content and superior gelatinization properties that enhance sugar release during fermentation [2]. Physicochemical parameters such as chalkiness degree [41], amylose content [42], and grain structure significantly impact wine quality [43,44]. Among rice types, round-grain glutinous rice, with lower amylose, promotes microbial utilization, boosting fermentation efficiency and flavor [29]. The amylopectin-to-fat ratio is a key brewing indicator; a ratio exceeding 15 can increase sensory scores by over 30% [45]. Japonica glutinous rice, for example, shows a higher short-chain amylopectin proportion, enhancing saccharification efficiency by 25% under 30 °C fermentation [29]. Each 1% increase in amylopectin content elevates esterase activity, promoting aromatic ester accumulation [46]. However, recent research challenges the assumption that all glutinous rice outperforms non-glutinous varieties, highlighting the importance of specific varietal characteristics [47].

Lipid and protein contents in rice also play crucial roles [48,49]. Moderate lipid levels promote ester accumulation and aroma intensity, while excessive lipids inhibit yeast metabolism and cause off-flavors [50]. Protein composition affects amino acid release, influencing Maillard reactions and flavor. Modern breeding technologies, like CRISPR-Cas9 gene editing, are creating new rice varieties with optimized amylopectin and lipid levels, improving fermentation kinetics and flavor [51,52]. Diversifying raw materials, including millet and black rice, is on the rise to meet consumer demands. Future research will focus on genome-wide selection models to precisely match rice properties with microbial fermentation, driving the industry’s shift towards a molecular-level design [2].

### 3.2. Water Quality

Water quality profoundly impacts rice wine characteristics. Regional variations in mineral composition and trace element content directly affect microbial metabolism during fermentation and final product taste. Geographic indication product specifications often impose strict water source requirements. For instance, Shunde red rice wine mandates the use of “pollution-free water from the Xijiang and Beijiang river systems within the designated production area, complying with national drinking water standards [53].” Traditionally, premium water sources are believed to supply essential trace elements that enhance microbial growth and foster unique regional flavors.

Ancient Chinese brewing practices favored water collected post-winter Winter Solstice, when total hardness ranges between 8–12° dH (moderately soft), creating an optimal calcium-magnesium ion gradient for “ion channel effects [54,55,56].” This water increases starch granule swelling by 18%, accelerates β-glucanase peak activity by 2 h, and enhances cell wall polysaccharide breakdown to release more fermentable sugars [57,58]. Modern spectroscopic analysis reveals that winter water contains 23% fewer nano-scale water clusters (average diameter 0.8 nm) compared to with summer water [57]. These smaller clusters penetrate starch’s dense layers more effectively. Combined with winter temperatures (8–12 °C), this promotes a 15% increase in amylopectin helix unwinding, exposing more enzymatic cleavage sites [59]. These seasonal physicochemical differences validate the ancient wisdom of “making qu in summer and brewing in winter [59].”

Contemporary brewing industries employ biomimetic strategies to replicate winter water characteristics: Reverse osmosis-ion recombination technology precisely controls Ca^2+^/Mg^2+^ molar ratios at 2.1:1, supplemented with 0.02% nano-silica sol, achieving 97% of traditional winter brewing’s saccharification efficiency [60,61]. This not only supports modernization but also confirms the profundity of ancient hydrological knowledge.

### 3.3. Raw Material Processing

Traditional rice wine production begins with soaking glutinous rice for 6–10 h to achieve full hydration for subsequent steaming. Unlike conventional rice cooking, steaming preserves grain integrity and prevents excess water absorption. Maintaining the cooled rice at ambient temperatures is crucial for microbial activity since because excessive heat can inhibit or kill yeast and molds [62].

Processing methods significantly shape flavor profiles. In traditional practices, soaking and steaming are pivotal steps influencing fermentation outcomes. Studies show prolonged soaking increases biogenic amine content, posing potential health risks [63,64]. However, intermittent superheated steam gelatinization can effectively replace traditional soaking-steaming. Researchers have also developed a “triple-water-spray steaming” technique [65,66]: First spray: 150 mL/kg of 50 °C water, steamed for 7 min. Second spray: 150 mL/kg of >80 °C water, steamed for 8 min. Third spray: 150 mL/kg of >80 °C water, steamed for 10 min. This method increases ester compound content and significantly improves taste. The supplementation of total acid by *Lactobacillus*, as an alternative to the rice soaking process, led to a 27.16% decrease in the concentration of the harmful biogenic amines, thereby enhancing the safety of the Chinese rice wine and improving its quality [63].

Advancements in technology have introduced novel pretreatment methods like expansion technology and liquefaction, enhancing saccharification efficiency and shortening fermentation cycles [39,67,68,69,70]. These innovations underscore the dynamic evolution of rice wine production, blending tradition with cutting-edge science.

## 4. Technological Regulation of Rice Wine Quality

### 4.1. Traditional Techniques vs. Modern Innovations

Traditional rice wine brewing involves steps such as rice washing, soaking, steaming, cooling, qu inoculation, and fermentation (Figure 1). These seemingly simple procedures embody profound traditional wisdom and empirical knowledge. For instance, soaking durations vary seasonally: 5 h in summer versus 10–20 h in winter until grains crumble easily [71]. Steaming requires precise heat control to avoid undercooked or mushy rice, while cooling demands optimal temperatures (hand-tested warmth) [72].

Modern technological advancements have introduced innovations. Zhu et al. found that vacuum soaking can effectively shorten the soaking time of rice in CRW production, reduce the brewing cycle, and at the same time, does not affect the quality of the rice wine [73]. Li et al. proposed that enzymatic extrusion-pretreated broken rice, using α-amylase to break down starches into simpler sugars, has a higher fermentation rate and efficiency during simultaneous saccharification and fermentation (SSF). This process also results in significantly higher amino acid content compared to with traditional methods, indicating that enzymatic extrusion pretreatment is a feasible alternative for the production of Chinese Rice rice wine (CRW) [39]. Wei et al. presented an innovative brewing technology, discarding the rice soaking process, by adding lactic acid bacteria to make up for total acidity [63]. Zhao et al. found that secondary fermentation with dry yeast, including *Saccharomyces cerevisiae* and commercial blends, reduces sweetness and acidity while increasing alcohol content, thus improving taste [74]. Additionally, novel pretreatment methods like expansion technology and liquefaction improve saccharification efficiency and shorten fermentation cycles, bridging tradition with modern science [39,67].

### 4.2. Fermentation Conditions and Parameter Control

The fermentation process in rice wine production involves several stages. Initially, rice is steamed and inoculated with qu, which contains a diverse microbial community. These microorganisms, including molds like Aspergillus oryzae and Rhizopus species, break down the starches into simpler sugars. Subsequently, yeasts such as Saccharomyces cerevisiae convert these sugars into ethanol and carbon dioxide, producing alcohol. Additionally, bacteria like Hansenula anomala contribute to the development of unique flavors and aromas. This coordinated activity of different microbial species results in the characteristic profile of rice wine.

Table 1 summarizes critical factors influencing rice wine fermentation, their impacts, and optimization strategies to enhance quality and flavor consistency.

#### 4.2.1. Temperature Control: Decisive Factor in Fermentation Quality

Temperature serves as the pivotal control parameter in rice wine fermentation, governing microbial community succession and metabolic pathways through thermodynamic mechanisms. Studies demonstrate that the 28–30 °C range constitutes the optimal synergistic window for *Rhizopus oryzae* and *Saccharomyces cerevisiae*, during which saccharification enzyme activity peaks and ethanol conversion efficiency reaches its maximum (0.48 g/g·h) [62]. Under constant 30 °C conditions, amylopectin degradation rates and total ester content increase significantly, forming the characteristic “cereal-aromatic–-ester bouquet” flavor profile [75].

Deviation from this thermal optimum markedly impacts quality. Suboptimal temperatures below 25 °C induce lactic acid accumulation and residual sugar retention, yielding a sensory profile described as “sour-astringent with muted complexity [76].” Conversely, temperatures exceeding 33 °C provoke yeast cellular stress and disproportionate fusel alcohol synthesis, manifesting as pungent harshness and undesirable browning [77]. To mitigate these effects, industrial production employs gradient temperature modulation.

Saccharification phase: held at 30 °C to maximize enzymatic activity;

Primary fermentation: gradually lowered to 28 °C to balance metabolic rates.

Aging phase: further reduced to 25 °C to stabilize flavor compounds.

Precision temperature control (±0.3 °C) enhances flavor consistency and elevates sensory scores by 12–15%, providing scientific validation for modernizing traditional techniques [69]. This thermal engineering approach exemplifies how empirical wisdom and thermodynamic principles converge to optimize biochemical transformations in contemporary fermentation systems.

#### 4.2.2. Fermentation Duration: Temporal Dynamics of Flavor Formation

The fermentation time window of rice wine is dynamically regulated by temperature and raw material characteristics, forming a flavor generation system. At 30 °C, yeast alcohol dehydrogenase (ADH) activity peaks at 0.85 U/mL, compressing summer fermentation to 18–24 h, while winter temperatures (10–15 °C) reduce amylase activity by 40%, requiring 3–7 days of saccharification-fermentation [78]. A rice-water ratio of 1:1.6 stabilizes alcohol at 14.5% vol ±0.3, but ratios >1:2 dilute esters by 35% [79]. Modern processes use staged control: 48-h *Aspergillus oryzae* saccharification (α-amylase 145 U/g) reduces acetaldehyde by 28%, composite qu (Angel sweet qu + Baijiu qu at 0.6‰ + 5.3‰ dosage) controls higher alcohols at 220–340 mg/L. Aged at 5 °C for 36 h, this process boosts total esters to 2.1 g/L and achieves a 3-log reduction in bacterial load, indicating significant control over spoilage bacteria [80]. This integrates biochemical precision and process engineering for modern rice wine production.

#### 4.2.3. Qu and Yeast Dosage: Drivers of Fermentation Kinetics

The amount, type, and combination of qu cultures (e.g., traditional qu or commercial yeasts) are critical for rice wine fermentation. However, claims of “optimal” dosages (e.g., 1.5% sweet qu, 1% yeast) lack universal validation across different brewing scales and rice varieties [81]. While homebrewing suggests 3–4 g qu/500 g glutinous rice with temperature adjustments, this ignores microbial strain variability and substrate composition [47]. Claims that fixed inoculation ratios (e.g., 10% *Hansenula anomala*, 4% *Saccharomyces cerevisiae*) guarantee “high-quality” profiles (ethyl acetate 0.465 g/L) oversimplify complex microbial interactions, as flavor outcomes depend on dynamic metabolic networks [82]. The assertion that qu amounts must increase by 5 °C/decrease in home systems is mechanistically vague, lacking data on how temperature actually modulates enzyme activity vs. microbial viability [83]. Such models often overlook stochastic factors in small-scale setups, limiting their predictive utility for industrial applications.

#### 4.2.4. Raw Material Processing: Foundation of Quality

The selection and processing of raw materials are pivotal for rice wine quality, profoundly influencing fermentation and final product characteristics. Glutinous rice with high amylopectin-to-amylose ratios (higher amylopectin content) enhances wine body richness and sensory quality, while elevated fat content deteriorates palatability, underscoring the need for low-fat, high-amylopectin rice varieties [84].

The processing of raw materials proves equally crucial for optimal fermentation outcomes. must be soaked thoroughly until easily crushable by hand, ensuring complete hydration and preventing undercooking that could impair fermentation efficiency [85]. Subsequent steaming requires precise control to achieve a “cooked yet firm” texture, creating ideal conditions for microbial activity. Processing innovations further demonstrate significant impacts: when rice is ground into a paste prior to steaming, improved mash dispersion and liquefaction lead to a 9.31% increase in alcohol content [86]. Similarly, the combination of milling and saccharification techniques enhances alcohol yield by 8.61% compared to traditional cooked-paste fermentation [87]. These findings collectively emphasize that both the inherent properties of rice and carefully optimized processing methods critically determine fermentation performance and final product quality.

#### 4.2.5. pH and Acidity: Metabolic Gatekeepers

pH is an important environmental factor that affects rice wine fermentation [88]. It changes how microbes grow and what metabolic pathways they use. Yeast grows best when pH is between 3.0 and 7.5, with the ideal range being from 4.5 to 5.0. In practice, fermentation usually keeps pH between 5 and 7. Controlling acidity helps stop bad microbes from growing and helps good microbes work better. For example, setting pH to 3.3 before fermentation creates good conditions for fermentation [47].

Acidity also changes the flavor of rice wine. The right amount of acid makes the wine taste less bitter and more complex, with a better aftertaste. But too much organic acid makes the wine taste harsh. Studies show that controlling pH gives clear benefits. Adding 0.3% citric acid to set the starting pH to 4.2 ± 0.2 makes lactic acid bacteria grow much better than contaminants, changing their ratio from 1:1.5 to 8:1. It also increases ethyl acetate production by 55% [89]. In *Pueraria lobata* rice wine, keeping pH at 4.5, using a 1:1.8 material-to-liquid ratio, and fermenting for 5 days keeps 78.3% of puerarin and gives a sensory score of 89 [90]. This proves pH is crucial for managing microbes, enhancing flavor, and keeping nutrients in rice wine production.

#### 4.2.6. Substrate-to-Liquid Ratio and Hydration Management

The raw material-to-water ratio significantly influences rice wine fermentation efficiency and product quality. Research indicates optimal ratios vary considerably, ranging from 1:1 (water-to-material by weight) to 1:2.5 (by volume) or 80% water content (by volume), demonstrating the need for context-specific optimization [91]. In home fermentation practices, a commonly observed ratio yields approximately 900 g of rice wine from 500 g of rice, maintaining appropriate flavor concentration.

Water content exhibits dual effects on fermentation dynamics. Insufficient hydration restricts microbial metabolic activity and reduces fermentation efficiency, while excessive dilution diminishes flavor intensity and alcohol concentration [92]. This parameter further interacts with starch gelatinization, enzymatic activity, and mass transfer processes, requiring precise adjustment to balance microbial substrate accessibility with nutrient retention.

Optimal water ratio determination requires comprehensive consideration of multiple factors. Raw material characteristics, microbial physiological requirements, and desired sensory attributes collectively influence the ideal ratio [60]. These principles remain consistent across production scales, from artisanal practices to industrial manufacturing, with the common objective of achieving efficient fermentation while preserving product quality attributes.

#### 4.2.7. Hygiene and Contaminant Control

Hygiene critically impacts fermentation quality and product safety by preventing microbial contamination. Research indicates fermentation vessels require thorough cleaning, minimally involving scalding with boiling water to eliminate oils, raw water residues, and reduce microbial load. Production must exclusively employ cooled boiled water to prevent contamination [47]. Contact with oils or raw water should be strictly avoided as these may induce white mold growth, black spot formation, or discoloration. Rigorous hygiene and microbial control ensure smooth fermentation and stable rice wine quality.

#### 4.2.8. Additional Influencing Factors

In addition to the main factors mentioned above, there are several other factors that can also affect the fermentation process and quality characteristics of rice wine. For instance, the level of dissolved oxygen has a significant impact on microbial growth and metabolism [93]. Research has found that appropriately aerating the mixture at the beginning of fermentation to increase the dissolved oxygen content can promote microbial growth during the early stages of fermentation, as well as the generation of relevant enzymes and the initial transformation of raw materials [77]. Moreover, the stirring conditions are also an important influencing factor. Implementing a fermentation regimen that alternates between stirring and resting (i.e., stirring-resting-stirring) can not only enhance the activity of relevant enzymes during the early fermentation stage and improve the saccharification and fermentation capabilities, but also increase the yield of alcohol by 5.04% and shorten the fermentation cycle by approximately 2 to 3 days [94].

## 5. Microbial Roles in Rice Wine Fermentation

### 5.1. Microbial Diversity and Its Dynamic Changes

In traditional rice wine fermentation, the interplay of diverse microbial communities is crucial for determining alcohol content, flavor profiles, and overall quality. Molds, such as *Rhizopus* and *Aspergillus*, initiate the process by secreting saccharification enzymes that break down starch into fermentable sugars [95]. These molds lay the groundwork for subsequent fermentation steps by providing a rich supply of glucose.

*Saccharomyces cerevisiae* then takes center stage, leveraging anaerobic respiration to convert glucose into ethanol, thereby driving alcohol production [96]. This yeast’s high alcohol tolerance and efficient fermentation capabilities are vital for achieving the desired alcohol levels in rice wine.

Lactic acid bacteria (LAB), including *Lactobacillus* and *Pediococcus*, contribute to the complexity of rice wine by producing lactic acid. This not only lowers the pH but also serves as a precursor for the formation of ethyl lactate, a key aroma compound that imparts a creamy, buttery note to the final product [76]. Meanwhile, acetic acid bacteria generate ethyl acetate, adding fruity and floral nuances to the bouquet [97].

High-throughput sequencing has unveiled the intricate dynamics of microbial succession throughout fermentation. In the early stages, fungi such as *Saccharomyces cerevisiae*, *Saccharomycopsis fibuligera*, and *Rhizopus oryzae* dominate, focusing on saccharification [98]. As fermentation progresses, bacterial communities like *Citrobacter* and *Exiguobacterium* rise to prominence, influencing acid and ester formation [99]. In the later stages, genera like *Staphylococcus*, *Pediococcus*, and *Weissella* take over, fine-tuning the flavor profile through their metabolic activities [60].

This sequential microbial succession-from molds to yeasts and finally to bacteria-facilitates a cascade of biochemical transformations. Molds break down complex carbohydrates, yeasts convert sugars into alcohol, and bacteria modulate the acid and ester content. Collectively, these microbial communities orchestrate the conversion of raw materials into a complex, aromatic, and flavorful rice wine, ultimately shaping its sensory characteristics.

By elucidating the roles of each microbial community, we gain deeper insights into the fermentation process, paving the way for targeted interventions to enhance rice wine quality and consistency.

### 5.2. Regional Differences in Microbial Communities

Geographically distinct rice wines demonstrate significant microbial community variations. Comparative analysis reveals that while both Yichang City and Suzhou City rice wines complete fermentation within two days, their microbial compositions differ substantially [100]. Suzhou samples are predominantly colonized by *Saccharomycopsis* and *Rhizopus* fungi, whereas Yichang specimens contain *Rhizopus*, *Wickerhamomyces anomalus*, *Saccharomyces cerevisiae*, *Mucor indicus*, and *Mucor microsclerotianus* as core fungal species. These geographical variations presumably originate from localized environmental microbiota, indigenous fermentation practices, and ingredient characteristics, ultimately defining each product’s distinctive organoleptic properties. Research further indicates bacterial diversity consistently surpasses fungal diversity, implying bacteria may contribute more substantially to rice wine fermentation than conventionally assumed [26,101].

### 5.3. Climate Conditions and Microbial Ecology

Geographical and climatic factors significantly determine microbial composition, consequently affecting rice wine quality. Despite sharing subtropical humid monsoon climates, Enshi (Hubei) and Dazhu (Sichuan) demonstrate distinct microbial profiles in their rice wines. While both regions show Ascomycota and Mucoromycotina dominance at the phylum level, key genus-level differences emerge: Enshi’s microbiota is characterized by *Saccharomycopsis*, *Rhizopus*, and *Amylomyces*, whereas Dazhu’s features *Rhizopus*, *Saccharomycopsis*, *Saccharomyces*, and *Wickerhamomyces* as predominant fungal genera [102,103].

The flavor profiles are significantly influenced by regional variations in microbial composition. For instance, rice wines from Xiaogan and Dazhou show divergent fungal communities, driven by low-abundance microbial groups. While *Rhizopus* and *Saccharomyces* are common dominant genera, *Clavispora* is unique to Xiaogan and *Candida* to Dazhou. These genus-specific microbes, particularly *Candida* and *Clavispora*, are closely linked to aromatic compound production, playing a critical role in shaping each region’s characteristic flavor profiles [104].

### 5.4. Microbial Interactions in Fermentation Qu

The unique brewing system of rice wine centers on the “fermentation qu” technology, a bioengineering technique originating in China that has profoundly influenced the development of fermented foods worldwide. The fermentation qu (also known as starter, koji, Jiuyao, etc.) holds multiple values in rice wine production [47].

**A Carrier of Diverse Microbial Consortia:** It provides a rich source of dominant microbial strains for subsequent fermentation. Modern microbiology has isolated hundreds of functional strains from qu.

**Synergistic Saccharification and Alcohol Fermentation**: Microorganisms in qu, such as *Rhizopus and Mucor*, secrete saccharification enzymes that convert rice starch into fermentable sugars, enabling simultaneous saccharification and fermentation.

**Production of Flavor Precursors:** Enzymes produced by microbial metabolism in qu, such as esterases and proteases, catalyze the formation of unique amino acid profiles and aromatic compounds, contributing to the complex flavor profile of rice wine.

**Simplified Production Processes:** Solid-state qu production ensures microbial viability, facilitates long-term storage, and is adaptable for industrial applications.

**Shaping Regional Flavor Characteristics:** The unique temperature and humidity conditions of different regions give rise to distinct types of qu, such as Xiaoqu (small Qu), Red Qu, and Wheat Qu. Regional rice wine styles are characterized by a diverse flavor system formed by these microbial variations.

The characteristic flavor profile of Chinese rice wine is determined by complex microbial communities and their metabolic functions. The fermentation process predominantly involves molds (primarily *Rhizopus* spp.), yeasts, and lactic acid bacteria, which exhibit facultative anaerobic heterotrophic metabolism [12,26]. Scientific investigations have identified four principal fermentation stages: acidification, saccharification, alcoholization, and esterification. *Rhizopus* species contribute critically during saccharification through the enzymatic secretion of α-amylase, β-amylase, and glucoamylase, catalyzing the sequential degradation of amylose and amylopectin into dextrins, maltose, and ultimately glucose [105]. *Saccharomyces* spp. dominate the alcoholization phase, mediating the anaerobic conversion of glucose to ethanol and carbon dioxide [106]. Concurrently, lactic acid bacteria and acetic acid bacteria generate organic acids [107] (e.g., lactic acid, acetic acid) that subsequently serve as essential precursors for ester synthesis, profoundly shaping the final aromatic characteristics of the product. Table 2 presents the principal factors influencing rice wine fermentation and their roles.

The flavor profile of rice wine is substantially influenced by microbial interactions during fermentation [6,108]. For example, *Aspergillus niger* co-fermented with *Saccharomyces cerevisiae* doubles polygalacturonase production, while *Candida utilis* inhibits *S. cerevisiae* aerobically but not anaerobically. A comprehensive understanding of rice wine flavor requires analysis of microbial interactions, metabolic correlations, and environmental impacts. Genetic engineering approaches, including ethanol-tolerant mutant strains (e.g., F23), provide novel microbial resources for quality enhancement [109].

### 5.5. Impact of Seasonal Factors on Fermentation

Traditional Chinese rice wine fermentation is characteristically performed in open environments, resulting in increased microbial community complexity and enhanced flavor compound diversity. The fermentation process has been demonstrated to be significantly influenced by seasonal variations, with superior quality and more balanced flavor profiles being consistently observed in wines fermented during the Winter Solstice period [54,55]. This phenomenon may be attributed to the selective proliferation of psychrotolerant microorganisms under low-temperature conditions and their subsequent contributions to flavor development.

## 6. Major Compounds in Rice Wine and Their Formation Mechanisms

Table 3 provides a comprehensive summary of the major compounds in rice wine. These compounds are formed during fermentation and interact with each other to ultimately determine the flavor, texture, and quality of rice wine. Different types of rice wine vary in the types and concentrations of these components, thereby presenting distinct flavor profiles [110].

### 6.1. Classification of Major Flavor Compounds

The flavor of Chinese rice wine consists of volatile and non-volatile compounds, identified through techniques like GC-MS (gas chromatography–mass spectrometry) and GC-IMS (gas chromatography–ion mobility spectrometry). Volatile compounds dominate the aroma profile, including higher alcohols (e.g., isoamyl alcohol), esters (e.g., ethyl acetate), organic acids (e.g., lactic acid), aldehydes, ketones, and pyrazines [47]. For instance, Shaoxing rice wine contains 64 volatile compounds, while buckwheat rice wine has up to 107, including unique pyrazines. Higher alcohols provide alcoholic notes but require balance to avoid unpleasant odors [111]. Esters, the primary aromatic components, contribute fruity and floral nuances, formed by yeast esterases in late fermentation. The flavor profile of rice wine is governed by a delicate balance of various chemical constituents. Organic acids play a crucial role in modulating acidity and flavor complexity, yet excessive concentrations result in undesirable sourness. Aldehydes and ketones contribute to aromatic diversity, though their levels require precise regulation to avoid harsh sensory characteristics. Pyrazines, particularly prevalent in premium rice wine varieties, generate distinctive roasted and nutty flavor notes through high-temperature microbial metabolic processes.

Beyond volatile compounds, rice wine contains significant non-volatile components, including polysaccharides, phenolic compounds, amino acids, and mineral elements [60]. These constituents collectively influence mouthfeel, nutritional value, and product stability. Polysaccharides contribute to viscosity enhancement, while phenolic compounds provide antioxidant properties. The ultimate flavor composition is determined by multiple factors: raw material selection, fermentation parameters, microbial ecology, and environmental conditions. Strategic optimization of these variables enables customized flavor development to meet market demands. Future investigations should focus on elucidating the fundamental mechanisms of flavor formation to facilitate technological innovation and quality improvement in rice wine production [60].

### 6.2. Sources and Formation of Flavor Compounds

The complex flavor profile of rice wine emerges through multiple biochemical pathways involving raw materials, fermentation qu, microbial metabolic activities, and chemical transformations during the brewing process [112]. Higher alcohols, including propanol, isobutanol, and isoamyl alcohol, constitute essential flavor components, with their biosynthesis predominantly occurring during the initial 48- h fermentation phase via yeast-mediated Ehrlich pathways. These compounds are derived from precursor amino acids, including valine, thatwhich gives rise to isobutanol and leucine that leads to isoamyl alcohol, as well as glucose metabolism that produces phenylethanol [6]. Their production kinetics are closely correlated with nutrient utilization. Strategic interventions such as temperature modulation or nutrient regulation can effectively mitigate excessive accumulation and associated off-flavors.

Esters, the principal aromatic constituents, are synthesized through enzymatic esterification catalyzed by yeast-derived esterases, wherein carboxylic acids and alcohols combine to form volatile compounds. The main ethyl esters—ethyl acetate and ethyl lactate, formed from acetic acid with ethanol, and lactic acid with ethanol respectively—impart distinct fruity and floral aromas. It is worth noting that these esters are derived not only from alcohol-acid reactions but also through the action of microbial esterases. Their concentration exhibits a positive correlation with fermentation duration, while process optimization through controlled temperature elevation or acid supplementation can enhance esterification efficiency [112].

The acid profile, primarily comprising lactic and acetic acids, plays a dual role in flavor development. These compounds are generated through microbial metabolism (*Lactobacillus* spp. converting glucose to lactic acid; acetic acid bacteria oxidizing ethanol to acetic acid), with production peaking during later fermentation stages [113]. While serving as crucial acidity regulators and ester precursors, their concentrations require precise control to prevent sensory imbalance—excessive levels impart harsh sourness, whereas optimal amounts contribute to flavor complexity. Future research into these mechanisms will provide a scientific basis for modernizing rice wine production and enhancing its quality.

Table 4 summarizes the major categories of flavor compounds in rice wine, their representative components, flavor characteristics, and roles in rice wine. Differences in the content and proportions of these compounds among different types of rice wine lead to distinct flavor profiles.

## 7. Factors Affecting the Quality and Flavor of Rice Wine

### 7.1. Key Aroma Compounds and Their Sources

Rice wine owes its characteristic fragrance to a diverse array of aroma compounds [114]. Esters, being the most abundant in both variety and quantity, are the primary contributors to its rich aroma. For example, ethyl lactate, presenting fruity and creamy notes, is formed through the combination of lactic acid (produced by lactic acid bacteria) and ethanol (generated by yeast) under the catalysis of acetyl-CoA. This ester not only adds a pleasant aroma but also contributes to the smoothness of the wine’s taste.

Another significant group is the alcohols, among which β-phenylethyl alcohol is typical. Endowed with a strong rose and creamy scent, it is produced during the fermentation process, mainly by yeast metabolism, and is a key factor in imparting the floral and sweet-creamy aroma to rice wine.

Volatile organic acids, such as acetic acid and lactic acid, also play a role. They are not only precursors for ester synthesis but also contribute to the overall flavor balance of the wine. The acetic acid provides a sharp, acidic note that, in moderation, enriches the flavor profile, while lactic acid adds a soft, rounded acidity that complements the sweetness of the wine.

### 7.2. Impact of Different Fermentation Conditions on Flavor Profiles

Fermentation temperature is a crucial factor [115]. Lower temperatures, as in traditional Winter Solstice brewing, slow down the fermentation rate [54]. This allows cold-tolerant microbes to thrive, leading to a more balanced production of flavor compounds. For instance, a lower temperature may result in a slower production of ethanol, giving more time for the formation of esters and other secondary metabolites, thus enhancing the complexity of the flavor. In contrast, higher temperatures accelerate fermentation, but may lead to a more rapid and less-controlled production of flavor compounds, potentially resulting in a less- complex flavor profile.

The oxygen availability during fermentation also has a profound impact. Anaerobic conditions are ideal for yeast fermentation to produce ethanol. Under such conditions, acetic acid bacteria are suppressed, preventing the over-production of acetic acid, which could otherwise spoil the wine’s freshness [77]. However, a small amount of oxygen in the initial stages of fermentation can stimulate yeast growth and metabolism, promoting the production of certain flavor-enhancing compounds. But, excessive oxygen exposure can trigger oxidation reactions, leading to the degradation of flavor compounds and the development of off-flavors.

### 7.3. Comparative Analysis of Flavor Notes in Traditional vs. Modern Production Methods

Traditional production methods often involve the use of earthenware vessels. These porous containers allow for a slow exchange of oxygen, promoting a gradual oxidation process. This slow oxidation contributes to the development of a more complex flavor over time, as the wine ages in the vessel. The earthenware may also absorb and release certain trace elements, further influencing the flavor [116]. For example, the slow oxygen permeation can lead to the formation of additional esters and aldehydes, adding depth and complexity to the flavor profile.

Modern production methods, on the other hand, often utilize stainless-steel equipment. These vessels provide a more controlled environment, minimizing oxygen exposure. As a result, the wine produced in stainless-steel tanks tends to retain more of its fresh, fruity, and floral aromas [117]. The absence of oxygen-mediated reactions preserves the primary fermentation-derived flavor compounds, presenting a cleaner and more straightforward flavor profile compared to the traditional counterparts. However, the lack of slow oxidation may mean that the wine lacks the deep, aged-like flavors that traditional methods can produce.

In conclusion, the quality and flavor of rice wine are intricately determined by a combination of factors related to aroma compounds, fermentation conditions, and production methods. A comprehensive understanding and precise control of these elements are essential for producing high-quality rice wine with consistent and desirable flavor profiles.

## 8. Modern Research and Technological Advancements

Contemporary scientific and technological advancements have transformed rice wine production through precision-controlled processes and targeted microbial management [2]. The industry has evolved from artisanal methods to automated systems utilizing computer-regulated fermentation parameters (temperature, humidity, aeration) alongside enzymatic treatments (ultrasound/microwave-assisted biocatalysts) to optimize saccharification efficiency. Strain development employs genetic modification to create yeasts with enhanced ethanol tolerance and flavor-modulating capabilities, though GMO use in traditional rice wine remains limited. Preselected strains adapted to stress conditions are more commonly utilized. Advanced bioreactors with continuous parameter tracking (sugar, pH) enable optimized fermentation kinetics, minimizing off-flavor formation while preserving delicate aromatic profiles through controlled low-temperature fermentation.

Nutritional enhancement strategies focus on bioactive compound enrichment (amino acids, B vitamins, phenolic acids) via tailored saccharification protocols and microbial consortia design. Flavor profiling utilizes advanced analytical platforms (GC-MS, HPLC-MS) to characterize volatile/non-volatile components and their microbial origins, facilitating precision optimization [29]. Digital transformation, incorporating IoT and machine learning algorithms, enhances process automation, formula refinement, and production standardization. These innovations collectively modernize rice wine manufacturing, achieving harmonization between traditional quality attributes and contemporary production demands through science-driven approaches.

## 9. Analytical Techniques for Food Safety Monitoring

Rice wine production involves critical safety considerations, particularly concerning ethyl carbamate (EC) and biogenic amines (BAs). EC, a potential carcinogen, forms through chemical reactions between ethanol and nitrogenous compounds (amino acids, urea, or amines) during fermentation and storage. Elevated temperatures and nitrogen-rich raw materials can accelerate its generation [118]. Effective mitigation strategies include maintaining fermentation temperatures at 20–25 °C [119], limiting urea addition [36], and selecting raw materials with lower precursor concentrations [120].

Biogenic amines (BAs), such as histamine, tyramine, and putrescine, are produced primarily through microbial decarboxylation of amino acids by lactic acid bacteria and yeasts. High concentrations of these compounds can induce adverse physiological effects [121,122]. Control measures include employing microbial strains with reduced amine-producing capacity, optimizing fermentation duration, and implementing targeted antimicrobial interventions [15].

Accurate analytical methods are essential for detecting and quantifying these compounds to ensure food safety. Common techniques include the following:

Gas Chromatography-Mass Spectrometry (GC-MS) is widely used for its high sensitivity and specificity. GC-MS allows for the identification and quantification of multiple biogenic amines in complex matrices, such as fermented beverages.

High-Performance Liquid Chromatography (HPLC) is useful for detecting and quantifying biogenic amines in various food products, including rice wine.

Ultra-High-Performance Liquid Chromatography (UHPLC) offers higher resolution and sensitivity compared to traditional HPLC, making it suitable for rapid and accurate determination of biogenic amines.

These analytical techniques are crucial for monitoring the levels of EC, BAs, and other unwanted compounds in rice wine, ensuring that the final product meets food safety standards.

## 10. Conclusions and Future Perspectives

Rice wine, a traditional Chinese fermented beverage, has become an important subject of multidisciplinary research owing to its intricate microbial ecosystem, distinctive flavor chemistry, and potential health-promoting properties. This review systematically examines fundamental research areas encompassing fermentation biochemistry, microbial dynamics, flavor biosynthesis, and physiological functionalities. The microbial consortia, recognized as pivotal determinants of product quality, have been extensively characterized through advanced molecular approaches. Flavor development mechanisms have been progressively deciphered through a comprehensive analysis of key aromatic compounds and their metabolic networks. Furthermore, the identified bioactive constituents substantiate rice wine’s functional food potential. Future investigations should prioritize elucidating microbial interactions, developing process control technologies, characterizing bioactive components, harmonizing traditional and modern methodologies, and designing customized qu cultures. The integration of empirical knowledge with contemporary scientific principles will advance rice wine production in terms of quality optimization, safety assurance, and functional food development.

## Figures and Tables

**Table 1:** Key factors influencing rice wine fermentation.

Factor	Specific Conditions	Effects	Optimization Strategies
Raw material	Rice variety, starch/protein content	Determines saccharification capacity and amino acid profile, affecting fermentation efficiency and flavor	Select high-starch, moderate-protein rice; optimize starch-to-protein ratio
Saccharification	Temperature (50–60 °C), enzyme type	Governs starch hydrolysis efficiency and sugar yield	Use high-activity amylases; maintain optimal temperature range
qu culture	Microbial diversity, alcohol tolerance	Impacts fermentation rate, alcohol content, and aroma compounds	Employ superior qu or alcohol-tolerant yeast strains
Temperature	Low (10–15 °C), medium (15–25 °C), high (25–35 °C)	Low temp: floral notes; medium: full-bodied; high: risk of fusel alcohols	Match temperature to style (e.g., low for light rice wine, medium for huangjiu)
Duration	Short (3–7 d), medium (10–15 d), long (>20 d)	Longer fermentation enhances flavor complexity but risks off-flavors	Extend moderately; monitor metabolite profiles
Oxygen	Aerobic/anaerobic phases	Aerobic: yeast proliferation; anaerobic: ethanol production	Controlled aeration to balance yeast growth and avoid acetobacter Acetobacter contamination
pH	3.5–4.5	Affects microbial activity; extremes hinder saccharification or invite spoilage	Adjust with food-grade acids (e.g., lactic acid)
Microbial ecology	Lactic acid bacteria, molds	Beneficial microbes suppress pathogens; contamination causes off-flavors	Maintain balanced microbiota; use antimicrobial qu strains
Vessel	Ceramic jars, wooden barrels, stainless steel	Influences oxidation and flavor exchange	Select based on target flavor (e.g., ceramic for oxidative aging)
Hygiene	Equipment cleanliness, air quality	Critical for microbial purity and product safety	Sterilize tools with boiling water; use filtered air systems

**Table 2:** Key factors affecting rice wine fermentation and their roles.

Fermentation Stage	Main Process	Key Reactions	Main Influencing Factors
Raw Material Preparation	Washing, soaking, and steaming of rice	Gelatinization of starch, improved enzyme hydrolysis efficiency	Rice variety, water quality, and steaming temperature
Saccharification Stage	Addition of fermentation qu or molds, and enzymatic hydrolysis of starch	Starch → Glucoseglucose, maltose	Temperature (50–60 °C), enzyme activity, and pH value
Early Fermentation (Aerobic)	Yeast proliferation, lactic acid bacteria regulating acidity	Lactic acid production by lactic acid bacteria, yeast proliferation	Appropriate aeration, temperature control (20–30 °C)
Main Fermentation (Anaerobic)	Yeast fermentation of sugars, ethanol, and flavor compound production	C_6_H_12_O_6_ → 2C_2_H_5_OH + 2CO_2_	Temperature (10–25 °C), fermentation time, and yeast activity
Post-Fermentation and Maturation	Flavor compound formation, wine maturation	Esterification, oxidation of alcohols, and flavor integration	Low-temperature storage, oxygen control, and time (1–6 months)
Filtration and Packaging	Separation of lees, clarification, bottling, and sealing	Removal of impurities, ensuring clarity of the wine	Filtration method, storage containers, and sealing conditions

**Table 3:** Classification and summary of major compounds in rice wine.

Compound Category	Representative Compounds	Main Source	Functions and Characteristics
Carbohydrates	Glucose, maltose, oligosaccharides	Starch hydrolysis by amylase	Provide substrates for fermentation, impart sweetness and viscosity to rice wine
Alcohols	Ethanol, isoamyl alcohol, propanol, and isobutanol	Fermentation of sugars by yeast	Affect alcohol content and body of the wine; some higher alcohols impart fruity or spicy notes
Organic Acidsacids	Lactic acid, acetic acid, succinic acid, and malic acid	Metabolism by lactic acid bacteria and yeast	Influence acidity and flavor balance; some organic acids enhance the umami taste of rice wine
Esters	Ethyl acetate, ethyl lactate, butyl acetate, and ethyl malate	Esters formed by microbial esterase activity, not just alcohol-acid reaction.	Confer fruity, floral, or dairy aromas, enhancing the complexity of rice wine’s bouquet
Aldehydes and Ketonesketones	Acetaldehyde, furfural, and 3-methylbutanal	Produced during fermentation and aging	Acetaldehyde imparts grassy or fruity notes, while furfural contributes caramel or nutty aromas
Phenolic Compoundscompounds	p-Coumaric acid, ferulic acid, and phenolic compounds	Metabolism of rice and microorganisms	Influence the color, bitterness, and aging flavor of rice wine
Nitrogen-Containing containing Compounds	Glutamic acid, alanine, glycine, and peptides	Degradation of rice proteins	Enhance umami and body, improve the nutritional value of rice wine
Other Trace trace components	Carbon dioxide, sulfides, and metal ions	Fermentation by-products and water sources	Affect the effervescence, mineral flavor, and stability of rice wine

**Table 4:** Classification and summary of representative flavor compounds in rice wine.

Compound Category	Representative Compounds	Flavor Characteristics	Functions
Alcohols	Ethanol, isoamyl alcohol, propanol, isobutanol	Fruity, spicy, alcoholic sensation	Affect alcohol content and enhance aromatic complexity
Esters	Ethyl acetate, ethyl lactate, butyl acetate	Fruity, floral, dairy-like	Confer aroma to rice wine and soften the palate
Organic acids	Lactic acid, acetic acid, succinic acid	Soft acidity, freshness, umami	Regulate acidity and enhance flavor balance and umami
Aldehydes and ketones	Acetaldehyde, furfural	Grass-like, fruity, caramel-like	Influence the body and aging flavor of the wine
Nitrogen-containing compounds	Glutamic acid, alanine, glycine	Umami, sweetness, full-bodied	Enhance the complexity of the wine and make the palate more rounded

## Data Availability

No new data were created or analyzed in this study.
